# Supplementary material for: Unravelling Heterogeneity of Amplified Human Amniotic Fluid Stem Cells Sub-Populations
Source: Cells. 2021 Jan 15;10(1):158. doi: 10.3390/cells10010158 (PMC7830644; doi:10.3390/cells10010158)
Supplement: Supplementary file 1 [file cells-10-00158-s001.pdf]

Unravelling Heterogeneity of Amplified Human Amniotic Fluid Stem Cells Sub-Populations

Francesca Casciaro [1,2,3], Silvia Zia [4], Mattia Forcato [5], Manuela Zavatti [1], Francesca Beretti [1], Emma Bertucci [6], Andrea Zattoni [7], Pierluigi Reschiglian [7], Francesco Alviano [8], Laura Bonsi [8], Matilde Yung Follo [2], Marco Demaria [3], Barbara Roda [7,*] and Tullia Maraldi [1]

1 Department of Biomedical, Metabolic and Neural Sciences, University of Modena and Reggio Emilia, Modena, 41124 Italy; francesca.casciaro3@unibo.it (F.C.); manuela.zavatti@unimore.it (M.Z.); francesca.beretti@unimore.it (F.B.); tmaraldi@unimore.it (T.M.)
2 Cellular Signalling Laboratory, Department of Biomedical and Neuromotor Sciences, University of Bologna, Bologna, 40125, Italy; matilde.follo@unibo.it
3 European Research Institute for the Biology of Ageing (ERIBA), University Medical Center Groningen (UMCG), University of Groningen, Groningen, 9713, The Netherlands; m.demaria@umcg.nl
4 Stem Sel srl., Bologna, Italy; silvia.zia@stemsel.it
5 Department of Life Sciences, University of Modena and Reggio Emilia, Modena, 41124, Italy; mattia.forcato@unimore.it
6 Department of Medical and Surgical Sciences for Mothers, Children and Adults, University of Modena and Reggio Emilia, Azienda Ospedaliero Universitaria Policlinico, Modena, 41124, Italy; emma.bertucci@unimore.it
7 Department of Chemistry "G. Ciamician", University of Bologna, Bologna, 40125 Italy; andrea.zattoni@unibo.it (A.Z.); pierluigi.reschiglian@unibo.it (P.R.)
8 Unit of Histology, Embryology and Applied Biology, Department of Experimental, Diagnostic and Specialty Medicine, University of Bologna, Bologna, 40125, Italy; francesco.alviano@unibo.it (F.A.); laura.bonsi@unibo.it (L.B.)
* Correspondence: barbara.roda@unibo.it; Tel.: +39-051-209-9450

Supplementary Figures

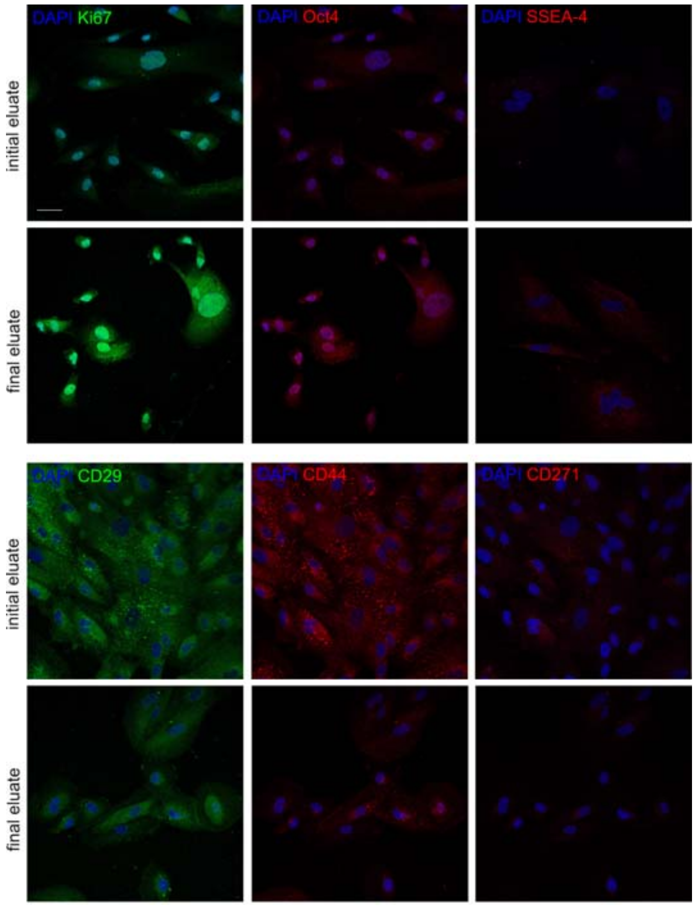

Figure S1. Evaluation of stemness markers of hAFSCs fractions. Representative confocal images (and magnifications) of initial or final eluates of hAFSCs labelled with DAPI (blue), anti-Ki67 and anti-CD29 in green and anti-Oct4, anti-SSEA-4, anti-CD44 and anti-CD271 in red. Bar = 10 μm.
